# Dispositional mindfulness profiles in pregnant women: relationships with dyadic adjustment and symptoms of depression and anxiety

**DOI:** 10.3389/fpsyg.2023.1237461

**Published:** 2023-09-06

**Authors:** Oiana Echabe-Ecenarro, Izaskun Orue, Esther Calvete

**Affiliations:** ^1^Department of Psychology, Faculty of Health Sciences, University of Deusto, Bilbao, Spain; ^2^Basque Health Service, Osakidetza, Zumaia, Spain

**Keywords:** mindfulness, pregnancy, depression, anxiety, dyadic adjustment

## Abstract

**Introduction:**

Pregnancy is a time of major transition that can be stressful for women. Dispositional mindfulness may protect individuals when they face stress. Recent studies have adopted a person-centered approach to examine the role of mindfulness by identifying subtypes of individuals based on their scores in five mindfulness facets. Latent profile analysis was used to identify different mindfulness profiles in a sample of pregnant women, and we explored the relationships between these profiles, depression and anxiety symptoms, and whether dyadic adjustment mediated these relationships.

**Method:**

A total of 535 women aged 18–45 years in their 26th week of pregnancy completed questionnaires regarding mindfulness, dyadic satisfaction and cohesion, and depression and anxiety symptoms.

**Results:**

Three profiles were identified: (1) low mindfulness (53.8%), (2) moderate mindfulness (34.3%), and (3) non-judgmentally aware (11.9%). The most adaptive profile was the non-judgmentally aware profile. Compared to the low mindfulness profile, the non-judgmentally aware profile and the moderate mindfulness profile were related to fewer symptoms of depression and anxiety, and these relationships were partly mediated by dyadic satisfaction.

**Discussion:**

These results suggest that analyzing each pregnant woman’s mindfulness profile can improve the prevention of and interventions for anxiety and depression.

## 1. Introduction

Pregnancy is a significant transition period for women, during which they prepare physically and psychologically for motherhood. During this transitional period, women must adapt to multiple changes, which may also include problems such as anxiety and depression. A systematic review of 21 studies with 19,284 participants found that 7.4% of the participants had depression in the first trimester, 12.8% in the second trimester, and 12% in the third trimester (Bennett et al., 2004). Further, a meta-analysis of 173 studies concluded that the rate of depression among pregnant women was 20.7% (Yin et al., 2021). Dennis et al. (2017) also conducted a meta-analysis and reported a prevalence of anxiety of 18.2% in the first trimester, 19.1% in the second trimester, and 24.6% in the third trimester.

Symptoms of anxiety and depression during pregnancy are predictors of negative consequences, including the recurrence of the same symptoms in the postpartum period (Heron et al., 2004; Vismara et al., 2021) and problems in the emotional and physiological development of the infant (Field, 2017a,b). Therefore, it is of great importance to identify factors that can reduce these symptoms and promote the wellbeing of pregnant women. In this study, we propose that dispositional mindfulness is one factor that can prevent women from experiencing symptoms of anxiety and depression.

*Mindfulness* is defined as bringing one’s attention to the experiences of the present moment and accepting them without judgment (Kabat-Zinn, 2003). Numerous studies have found that mindfulness is positively correlated with life satisfaction (Mattes, 2019) and negatively with psychological problems, including depression and anxiety (Tomlinson et al., 2018), particularly during pregnancy (Krusche et al., 2019). Furthermore, although numerous studies have included dispositional mindfulness in the form of a total score, factorial studies have revealed that it can be considered a multidimensional trait. The most commonly followed multidimensional model is that of Baer et al. (2006), who proposed a five-dimensional structure that is typically measured with their Five Facet Mindfulness Questionnaire (FFMQ): (1) observing (i.e., paying attention to internal or external experiences); (2) describing (i.e., using words to describe inner experiences); (3) acting with awareness (i.e., paying attention to the present moment); (4) non-judging of inner experiences; (i.e., not evaluating thoughts and feelings); and (5) non-reacting to inner experiences (i.e., allowing feelings and thoughts to come and go). Numerous studies have found that these five facets may play different roles in both the wellbeing of individuals and the development of psychological problems. For example, Carpenter et al. (2019) found that non-judging and acting with awareness correlated highly with affective symptoms, but describing and non-reacting correlated moderately with these symptoms and there was no significant relationship between these symptoms and the facet of observing. Similarly, in another meta-analysis, Prieto-Fidalgo et al. (2022) found that all facets except observing covaried with symptoms of anxiety and depression and only acting with awareness and non-reacting were longitudinally related to those symptoms. These results suggest that the observing facet functions differently from the other facets. In fact, several studies have found positive relationships between observing and the other facets and psychological problems (e.g., Rudkin et al., 2018).

Further, studies that have focused on samples of pregnant women have found that acting with awareness is associated with lower stress and depression (Mennitto et al., 2021) and acting with awareness and non-reacting with a more positive perception of childbirth (Hulsbosch et al., 2021). Moreover, in support of the relationship between mindfulness and symptoms of depression and anxiety during pregnancy, several studies have found that mindfulness-based interventions improve symptoms of depression and anxiety among pregnant women (for a review, see Shi and MacBeth, 2017; Krusche et al., 2018; Babbar et al., 2021).

In recent years, several authors have employed and recommended the use of person-centered techniques, such as latent profile analysis, to assess dispositional mindfulness (Pearson et al., 2015; Bravo et al., 2016). This type of analysis examines the scores for continuous variables (mindfulness dimensions) for each participant and identifies subsamples (profiles) from participants with similar response patterns. Most studies on mindfulness profiles suggest that there are homogeneous profiles (similar scores in the different mindfulness facets) as well as heterogeneous profiles (high and low levels in different facets), the latter of which are ordinarily either particularly non-judgmentally aware (i.e., high on non-judging and acting with awareness but low non-judging on observing) or judgmentally observing (i.e., high on observing but low on non-judging and acting with awareness) (Lecuona et al., 2022). For example, one of the first mindfulness studies that employed this approach (Pearson et al., 2015) identified four profiles in a sample of college students: (1) high mindfulness (i.e., high on all five facets); (2) low mindfulness (i.e., low on all five facets); (3) judgmentally observing; and (4) non-judgmentally aware. Several subsequent studies have identified the same four profiles (e.g., Bravo et al., 2016; Kimmes et al., 2017; Sahdra et al., 2017). However, other studies have also found a different number of profiles using the FFMQ. For example, in a large sample of adults, Zhu et al. (2020) found three profiles: (1) average mindfulness, (2) low-to-average mindfulness, and (3) high non-judgmentally aware. Further, in a study on adolescents, Calvete et al. (2020) also found a three-factor solution: (1) moderate mindfulness, (2) judgmentally observing, and (3) non-judgmentally aware.

Most of these studies found that distinct profiles led to different psychological outcomes (e.g., Bravo et al., 2016; Calvete et al., 2020). In general, the heterogeneous non-judgmentally aware profile and the homogeneous profile with high scores in all facets are more beneficial, while the judgmentally observing profile and that with low scores on all facets have the highest number of psychological problems. For example, Pearson et al. (2015) found that the judgmentally observing and low mindfulness groups had more negative emotional symptoms. Similarly, Zhu et al. (2020) found that individuals who were classified as non-judgmentally aware reported the lowest levels of depression and negative affect. To the best of our knowledge, although there are studies on the negative relationship between general mindfulness and depression and anxiety during pregnancy (e.g., McDonald et al., 2021), there are no studies on mindfulness profiles in samples of pregnant women; therefore, the relationship of these profiles with pregnancy-specific depression and anxiety remains unknown.

Another relevant question is the identification of the mechanisms through which mindfulness profiles can contribute to the psychological wellbeing of pregnant women. In this study, we propose that dyadic adjustment (satisfaction and cohesion) might partially explain the relationship between dispositional mindfulness and psychological adjustment. We expect that individuals who are more connected with themselves can have a better dyadic adjustment by improving communication, closeness, and self–other connectedness (Karremans et al., 2017). In fact, multiple studies have found evidence in favor of a positive relationship between dispositional mindfulness and dyadic satisfaction (e.g., Kappen et al., 2018; Morin et al., 2023). Two meta-analyses— McGill et al. (2020) and Quinn-Nilas (2020)—found an effect size of 0.27 and of 0.24, respectively, on the relationship between mindfulness and dyadic satisfaction. In a sample of 164 participants, Lenger et al. (2017) found that non-judging was the only mindfulness facet that was significantly related to dyadic satisfaction when all facets were introduced in the same model. In another study with 330 participants, Gobout et al. (2020) found that the facets of describing and non-judging were related to satisfaction in a path analysis. In a profile study, Kimmes et al. (2017) found that the high mindfulness profile and the non-judgmentally aware profile were significantly related to benign attributions in couple relationships. These results support the notion that different mindfulness profiles may differ in terms of how they are associated with perceptions in dyadic relationships.

In turn, these dyadic variables have been found to be related to symptoms of depression and anxiety. For example, Whisman et al. (2011) found a negative relationship between marital satisfaction and symptoms of depression and anxiety in a sample of pregnant women. Similarly, Alves et al. (2018) found that women with depression scored lower than women without depression in dyadic adjustment. Studies have also found a negative relationship between dyadic cohesion and symptoms of depression and anxiety (Abbas et al., 2019), although these studies did not specifically apply to pregnant women. Thus, in this study, we propose that dyadic satisfaction and cohesion may partially explain the relationships between mindfulness profiles and symptoms of depression and anxiety during pregnancy.

In summary, the principal aim of the present study was to analyze the association between dispositional mindfulness and symptoms of depression and anxiety in pregnant women and to test whether dyadic satisfaction and cohesion mediate this relationship. In this study, dispositional mindfulness was assessed using person-centered techniques (Pearson et al., 2015; Bravo et al., 2016) and we expected to find that a solution of three or four dispositional mindfulness profiles would adequately explain the mindfulness profiles of pregnant women. We hypothesized that profiles that were identified in previous studies as more adaptive (i.e., high in mindfulness and non-judgmentally aware) would be associated with greater dyadic cohesion and satisfaction and these variables, in turn, would be associated with fewer symptoms of depression and anxiety. In other words, we expected that dyadic satisfaction and cohesion would mediate the relationship between these profiles and the symptoms of depression and anxiety.

Our model considered past prenatal loss because it has been related to maternal depression and anxiety, particularly in the period immediately following pregnancy (Blackmore et al., 2011; Gower et al., 2023). Studies that compare levels of anxiety and depression in women with and without previous prenatal loss have revealed that levels of anxiety and depression are higher in women who have suffered prenatal loss (Smorti et al., 2021); therefore, it is important to control for this variable.

## 2. Materials and methods

### 2.1. Participants

The initial sample consisted of 586 pregnant women in their 26th week of gestation. Of these participants, 44 women indicated that they were not in a relationship and were eliminated from the analyses of this study. The resulting 542 women ranged in age from 18 to 44 years old (*M* = 33.39; *SD* = 4.42). The demographic information of the participants indicated that 90.5% were from Spain, 5.8% were from a South American country, 1.9% were from an African country, and the remaining 1.8% were from various European countries. This pregnancy was the first child for 74.4% of participants, the second for 20.7% of participants, and the third or greater for 4.9% of participants. For 86% of participants, the pregnancy had been planned, and 31.3% had experienced a previous prenatal loss.

### 2.2. Instruments

Dispositional mindfulness was measured using the FFMQ. This questionnaire contains 39 items and measures five facets: observing, describing, acting with awareness, non-judging, and non-reacting. The participants answered each item on a Likert scale ranging from 1 (never or very rarely true) to 5 (very often or always true). The Spanish version of the FFMQ has good psychometric properties (Cebolla et al., 2012).

The Edinburgh Postnatal Depression Scale (EPDS; Cox et al., 1987) was used to measure the participants’ symptoms of depression. This tool consists of 10 items with four response options that correlate with the increasing severity of the symptoms. The scale has obtained useful psychometric indicators for the pregnancy period in its Spanish validation (Vázquez and Míguez, 2019).

The seven-item Generalized Anxiety Disorder scale (Spitzer et al., 2006) was used to measure anxiety symptoms. It contains seven Likert-type response items, ranging from 0 (not at all) to 3 (nearly every day). The scale also obtained good psychometric indicators in its Spanish validation (García-Esteve et al., 2003). The Spanish version of this tool was recently validated for pregnant women (Soto-Balbuena et al., 2021).

The dyadic adjustment scale (Spanier, 1976) was used to measure the quality of the couple’s relationships. Although the tool contains four subscales (dyadic consensus, satisfaction, cohesion and affectional expression), this study used only dyadic satisfaction (10 items; score range of 0–50) and cohesion (five items; score range of 0–24) which were the focus of the study.

### 2.3. Procedure

This study was approved by the Clinical Research Ethics Committee of Euskadi and the University of Deusto. A total of 12 midwives from 12 different health centers in Basque Country were contacted. From among these, 10 agreed to collaborate and contacted the pregnant women at their health centers. Pregnant women filled out the questionnaires during their 26th week of pregnancy during their check-ups. All women were informed of the study and those who agreed to participate signed a written consent form. Thereafter, they were provided with the data collection questionnaire, which they filled out in an office at the health center. It took them 15–20 min to complete the questionnaire. They submitted the questionnaire anonymously. The researchers’ contact information was made accessible so that the participants could ask any questions they had.

### 2.4. Data analysis

The percentage of missing values in the study variables was 0.49%. Little’s Missing Completely at Random (MCAR) test was statistically non-significant [χ^2^(35) = 27, *p* = 0.813] and missingness was addressed by Full Information Maximum Likelihood (FILM). The distribution of the data was examined because, if the distribution is not normal or there are outliers, the results of the latent profile analysis may be biased (Spurk et al., 2020). The skewness and kurtosis indicators were adequate for all variables. In addition, multivariate outliers were checked by calculating the Mahalanobis distance for scores on the five dispositional mindfulness facets. Seven cases with *p*-values less than 0.001 were identified and discarded. Thus, the final sample size was 535.

Latent profile analysis (LPA) was employed in MPLUS 8.9 (Muthén and Muthén, 1998–2021) to explore participants’ profiles with regard to mindfulness facets. The criteria proposed in the literature to determine the optimal number of profiles was used (e.g., Nylund-Gibson and Choi, 2018; Spurk et al., 2020). For the estimation of the profiles, we used 7,000 random sets of start values, 300 iterations for each random start, and the 200 best solutions retained for final stage optimization. Initially, a single-profile LPA model was developed to serve as a comparative baseline for models with more than one profile. Thereafter, we increased the number of profiles by one and examined whether the resulting solutions were statistically and conceptually superior to the previous one (Nylund-Gibson and Choi, 2018). The following metrics were utilized to compare the models: the Bayesian information criterion (BIC), the Akaike information criterion (AIC), the sample size adjusted Bayesian information criterion (SABIC), the entropy index, the Adjusted Lo-Mendell-Rubin (LMR) test, the Bootstrapped Likelihood Ratio test (BLRT), and the mean posterior probabilities of the participants’ assignments to profiles. The BIC, AIC, and SABIC are approximate fit indices, and lower values indicate a superior fit (Nylund-Gibson and Choi, 2018). Higher entropy values suggest a better fit, and values approaching 0.80 or higher indicate a good classification of participants into profiles, although values between 0.60 and 0.80 are also considered adequate (see Spurk et al., 2020). The LMR test and the BLRT compare whether a solution of k profiles fits better than a solution of k-1 profiles. Mean posterior probabilities explain how well a given model classifies individuals into groups. Values of 0.70 or higher are considered adequate (Nagin, 2005). The number of participants within each profile were examined because profiles containing too few participants may not be replicated in other samples. Thus, additional profile solutions were discarded when the additional profile was of small size (e.g., less than 25 participants or <1% of the total sample; Spurk et al., 2020). In addition, the patterns of the results for the profiles were reviewed to ensure that they made theoretical sense and were similar to those obtained in previous studies.

To examine the relationships among the variables, the maximum likelihood estimation method was employed using MPLUS 8.9 (Muthén and Muthén, 1998–2021). Goodness of the model fit was assessed with the root mean square error of approximation (RMSEA), the comparative fit index (CFI), the Tucker-Lewis index (TLI), and the standardized root mean square residual (SRMR). CFI and TLI values of 0.90 or higher, RMSEA values lower than 0.06 and SRMR values lower than 0.08 usually indicate a good fit (Hu and Bentler, 1999; Little, 2013). Indirect associations between profiles and symptoms through couple relationships were tested via bootstrapping (*N* = 10,000 samples).

## 3. Results

Table 1 presents the means, SD, and Cronbach’s alphas for each study variable and correlations among them. The only facets that did not correlate with each other were observing and acting with awareness. Observing, describing, and non-reacting were positively related to both dyadic satisfaction and cohesion, but acting with awareness and non-judging were only related to dyadic satisfaction. All facets except observing were negatively related to depression and anxiety symptoms. In addition, dyadic satisfaction and cohesion were both negatively related to depression and anxiety symptoms.

**TABLE 1:** Means, standard deviations, and correlations among study variables.

	1	2	3	4	5	6	7	8	9	*M*	SD	Skew.	Kurt.
1. Observing	0.76									25.53	5.23	-0.21	-0.16
2. Describing	0.35**	0.87								28.84	5.33	-0.21	-0.18
3. Acting with awareness	-0.02	0.37**	0.86							29.35	5.37	-0.31	-0.06
4. Non-Judging	-0.13*	0.26**	0.45**	0.89						21.21	6.61	-0.38	-0.19
5. Non-Reacting	0.25**	0.45**	0.26**	0.32**	0.71					12.07	4.01	-0.15	0.44
6. Dyadic satisfaction	0.12*	0.21**	0.13*	0.11*	0.22**	0.76				41.31	5.32	-1.60	4.02
7. Dyadic cohesion	0.17**	0.22**	0.07	0.03	0.18**	0.46**	0.64			16.30	3.94	-0.41	0.22
8. Depression	-0.04	-0.34**	-0.35**	-0.53**	-0.42**	-0.39**	-0.14**	0.83		7.01	1.02	1.01	1.00
9. Anxiety	0.04	-0.25**	-0.32**	-0.48**	-0.33**	-0.32**	-0.10*	0.74**	0.86	12.07	0.83	0.86	0.40

Cronbach alphas for the study variables are shown in the diagonal. Skew. = Skewness; Kurt. = Kurtosis. *p < 0.05 and **p < 0.001.

Table 2 shows the LPA results. The optimal LPA model for the sample was the three-profile latent model. The LMR test *p*-value for the comparison between the two-profile and three-profile models was significant, but the comparison between the three-profile and four-profile models was not significant. The three-profile model substantially reduced the values for AIC, BIC, and SABIC compared to models with fewer profiles. The mean posterior assignment probabilities for profiles 1, 2, and 3 were 0.89, 0.89, and 0.94, respectively. Finally, the number of participants in each class was sufficient for all models (>25 participants and >1% of the total sample; Spurk et al., 2020).

**TABLE 2:** Results of the latent profile analyses.

	Number of profiles			
Fit statistics	1	2	3	4
LL	−3,793	−3,642	−3,571	−3,531
FP	10	21	32	38
AIC	7,606	7,326	7,206	7,138
BIC	7,649	7,416	7,343	7,300
SABIC	7,616	7,350	7,241	7,180
Entropy		0.68	0.76	0.71
LMR (*p*)		0.001	<0.001	0.075
BLRT (*p*)		<0.001	<0.001	<0.001
Sample size of each profile	P1 = 535	P1 = 340 P2 = 195	P1 = 273 P2 = 194 P3 = 68	P1 = 136 P2 = 62 P3 = 128 P4 = 209

P, Profile; LL, Model Log-Likelihood; FP, Free Parameters; AIC, Akaike Information Criterion; BIC, Bayesian Information Criterion; SABIC, the Sample-adjusted Bayesian Information Criterion; LMR(p), *p*-value for the adjusted Lo-Mendell-Rubin test; BLRT(p), *p*-value for the Bootstrapped Likelihood Ratio test.

**FIGURE 1:**
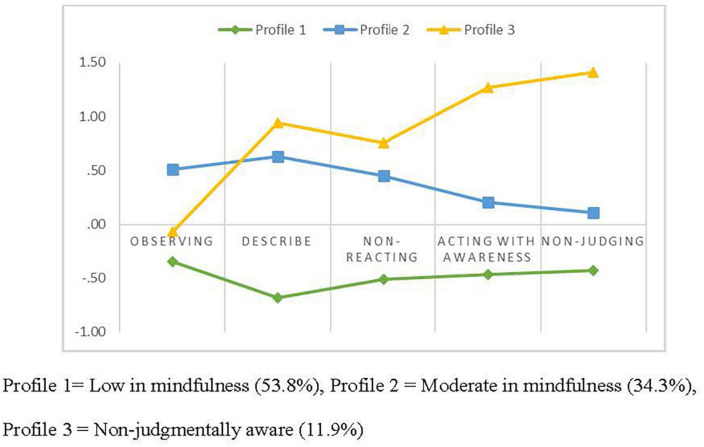
Three-profile solution in dispositional mindfulness.

After determining that, according to the empirical criteria, the three-profile solution was optimal, we examined this solution from the point of view of its content. The first profile was characterized by relatively low scores on all facets (low in mindfulness), the second profile was characterized by relatively moderate scores on all facets (moderate in mindfulness), and the third profile was characterized by the highest scores on acting with awareness and non-judgment and low scores on observing (non-judgmentally aware). These profiles are consistent with those obtained in previous studies (e.g., Zhu et al., 2020). Posterior probabilities were used to assign each participant to a single profile. Figure 1 shows the three profiles according to their z-scores for the five mindfulness facets. Table 3 presents the mean for each facet and the analysis of variance results, which were statistically significant for all facets. According to multiple comparisons (Bonferroni method, *p* < 0.05), the three profiles were statistically different from each other in all facets, except that there was no significant difference between profiles 1 (low in mindfulness) and 3 (non-judgmentally aware) in observing.

**TABLE 3:** Mean differences between profiles in mindfulness facets and model variables.

	Profile 1 low in mindfulness *n* = 273	Profile 2 moderate in mindfulness *n* = 194	Profile 3 non-judgmentally aware *n* = 68	*F*	*p*	η ^2^_p_
**Mindfulness facets**						
Observing	2.98_a_ (0.60)	3.53_b_ (0.47)	3.16_a_ (0.81)	49.87	<0.001	0.16
Describing	3.17_a_ (0.52)	4.04_b_ (0.39)	4.25_c_ (0.50)	260.89	<0.001	0.50
Acting with awareness	3.37_a_ (0.66)	3.82_b_ (0.41)	4.52_c_ (0.31)	131.81	<0.001	0.33
Non-Judging	3.18_a_ (0.77)	3.62_b_ (0.58)	4.68_c_ (0.22)	143.55	<0.001	0.35
Non-Reacting	2.78_a_ (0.48)	3.31_b_ (0.34)	3.48_c_ (0.67)	103.15	<0.001	0.28
Dyadic Cohesion	3.45_a_ (0.77)	3.61_ab_ (0.69)	3.77_b_ (0.76)	5.92	0.003	0.02
Dyadic satisfaction	4.30_a_ (0.52)	4.48_b_ (0.36)	4.55_b_ (0.48)	12.79	<0.001	0.04
Depression	0.93_a_ (0.47)	0.55_b_ (0.33)	0.33_c_ (0.27)	76.92	<0.001	0.22
Anxiety	1.90_a_ (0.57)	1.63_b_ (0.46)	1.33_c_ (0.31)	39.89	<0.001	0.13

^a, b, c^Means sharing a subscript in a row indicate means that are not significantly different from each other.

Table 3 also shows the differences across the profiles for the remaining study variables. The non-judgmentally aware group reported the lowest scores for symptoms of depression and anxiety. In addition, the moderate mindfulness group also scored significantly lower than the low mindfulness group in terms of depression and anxiety symptoms. Regarding couple variables, the non-judgmentally aware group scored higher than the low mindfulness group in dyadic cohesion. Finally, the non-judgmentally aware and moderate mindfulness groups scored significantly higher in dyadic satisfaction when compared to the low mindfulness group.

**FIGURE 2:**
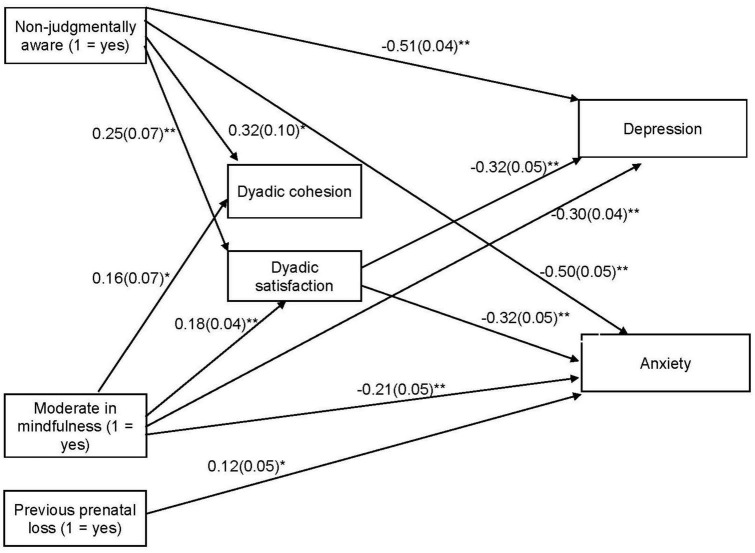
Mediational Model of the Association between Mindfulness, Dyadic Adjustment and Symptoms of Depression and Anxiety. Unstandardized coefficients are shown. Standard errors are in parentheses. *p < 0.05 and **p < 0.001.

Next, a path analysis was completed to examine the associations between the mindfulness profiles, dyadic factors, and psychological symptoms. The non-judgmentally aware and moderate mindfulness profiles were included as dummy variables (0–1) and compared with the low mindfulness profile. The model showed adequate fit indices [χ^2^(2,535) = 5.38, *p* = 0.068; RMSEA = 0.056 (90% CI = 0.00, 0.11); CFI = 0.995; TLI = 0.956; and SRMR = 0.020]. Figure 2 shows only the statistically significant paths. Both profiles were associated with greater dyadic cohesion and satisfaction and lower depression and anxiety symptoms. Consequently, satisfaction was associated with lower psychological symptoms. Previous prenatal loss was related to higher anxiety scores and depressive symptoms that were marginally significant. The results of the bootstrapping procedure indicated the significance of the indirect effect of both profiles on fewer symptoms of anxiety and depression through higher dyadic satisfaction: non-judgmentally aware with depression via satisfaction [95% CI: −0.134, −0.033]; non-judgmentally aware with anxiety via satisfaction [95% CI: −0.138, −0.033]; moderate mindfulness with depression via satisfaction [95% CI: −0.092, −0.029]; and moderate in mindfulness with anxiety via satisfaction [95% CI: −0.093, −0.030]. This model explained 32 and 20%, respectively, of the depression and anxiety scores.

## 4. Discussion

The main objective of this study was to examine the association between dispositional mindfulness profiles and symptoms of depression and anxiety in pregnant women and to determine whether this association was explained by indicators of relationship adjustment (satisfaction and cohesion). The results revealed that mindfulness profiles were associated with symptoms of depression and anxiety in pregnant women and that this was partially explained by the extent of dyadic satisfaction. The main results are discussed below.

Consistent with a growing line of research (e.g., Pearson et al., 2015; Bravo et al., 2016; Calvete et al., 2020), dispositional mindfulness was assessed using a person-centered approach. Through this, the profiles of pregnant women were identified according to their mindfulness facets. The results suggested a solution of three profiles as the most appropriate to describe the scores of these women in the following distinct mindfulness facets: low in mindfulness (53.8%), moderate in mindfulness (34.3%), and non-judgmentally aware (11.9%). The non-judgmentally aware profile scored significantly higher than the other profiles in all facets of dispositional mindfulness, except in observing. There were no significant differences in this facet between the low mindfulness and the non-judgmentally aware profiles, whereas the moderate mindfulness profile scored significantly higher than the remainder in this facet. The profiles identified in the present study among pregnant women are very similar to those found in the study by Zhu et al. (2020) in a sample of 1,727 adults in the Netherlands or those found by Bravo et al. (2018) in a sample of military personnel. However, most previous studies with adults have found four profiles. Specifically, most of them have identified, in addition to the three profiles that emerged in the present study, a fourth heterogeneous profile called “judgmentally observing,” with high scores for the observing facet and low scores on the non-judging of inner experience and acting with awareness facets (e.g., Pearson et al., 2015; Bravo et al., 2016). In this study, we did not find a judgmentally observing profile. It is important to consider that in previous studies, the judgmentally observing group was small; for example, it was 5.5% of the meditators’ sample and 12.87% of the non-meditators’ sample in the study by Bravo et al. (2016). It may be that we were unable to identify this profile in this study because we used a smaller sample. Another difference with respect to the studies that identified these profiles is that those studies included both men and women, while only women participated in the present study.

Interestingly, these profiles presented significant differences in dyadic cohesion, satisfaction, and psychological symptoms during pregnancy. The differences in the study variables according to the profiles were in line with what we expected and in accordance with previous studies (Pearson et al., 2015; Kimmes et al., 2017; Zhu et al., 2020). In general, these results suggest that the non-judgmentally aware profile showed the best psychological adjustment, while the most vulnerable group in terms of psychological functioning consisted of women who scored low in all mindfulness facets (low mindfulness profile). In addition, women who were classified as non-judgmentally aware scored lowest in symptoms of depression and anxiety, followed by women who were classified as moderate in mindfulness. Although we are not aware of previous studies on mindfulness profiles in pregnant women, these results are consistent with studies that have found that higher scores on facets, such as acting with awareness and non-reacting, are associated with fewer symptoms and greater wellbeing in pregnant women (van den Heuvel et al., 2015; Hulsbosch et al., 2021; Mennitto et al., 2021). Therefore, these results highlight the importance of cultivating these facets during pregnancy to increment wellbeing. In addition, the non-judgmentally aware and moderate mindfulness groups revealed better dyadic satisfaction than the low mindfulness group, and the non-judgmentally aware group scored significantly higher than the low mindfulness group in dyadic cohesion. These results are in line with what was found by Kimmes et al. (2017), who found that the high mindfulness profile and the non-judgmentally aware profile were positively associated with benign attributions for partner transgressions, which are closely related to couple satisfaction. These results of the present study extend previous knowledge on pregnant women and reveal that women with high scores, particularly in the facets of acting with awareness and non-judging, perceive their relationship with their partners as more satisfying and more cohesive.

Therefore, the results regarding the psychological adjustment of the three mindfulness profiles that emerged in this study indicate that the best scores for psychological adjustment among pregnant women were related to high scores for non-judging and awareness and low scores for observing. In fact, interestingly, at a correlational level, observing was not related to symptoms of depression or anxiety. This finding is consistent with previous studies that have found that this facet is frequently not related to psychological symptoms or it is positively associated with more psychological problems (Brown et al., 2015; Royuela-Colomer and Calvete, 2016; Rudkin et al., 2018); thus, this finding emphasizes the importance of the study of mindfulness profiles in a manner that considers the scores on the different facets rather than using a global score.

With regard to the path analysis, the results revealed that both the moderate mindfulness and non-judgmentally aware profiles were related to fewer depression and anxiety symptoms when compared with the low mindfulness profile and that this relationship was partially mediated by dyadic satisfaction but not dyadic cohesion. Thus, these results suggest that pregnant women who are mindful, particularly those who do not evaluate feelings and thoughts as good or bad (non-judging) and who do not operate automatically without paying attention (aware), perceive more satisfaction in their couple relationships and this satisfaction in their relationships leads to fewer symptoms of depression and anxiety.

This study has a few limitations. First, it has a cross-sectional design and, thus, we cannot ensure the directionality of the proposed relationships. It could be that dyadic adjustment and the symptoms of anxiety and depression lead to a certain profile of mindfulness. In fact, Gómez-Odriozola and Calvete (2020) found that the relationship between dispositional mindfulness and symptoms of depression was bidirectional. Similarly, it could also be that symptoms of anxiety and depression lead to dyadic adjustment, and not the other way around. In addition, it would be rather interesting to obtain data from women after childbirth to assess these relationships during pregnancy and postpartum. Second, all measures were self-reported by only one member of the couple. It would have been valuable to obtain measurements of, for example, the partners of the pregnant women and their levels of dyadic satisfaction and cohesion. Even so, we consider that the perception that the woman has of her dyadic adjustment and of her wellbeing during pregnancy is important beyond what people around her can assess. A third limitation of the study is the absence of a scale for measuring social desirability. Moreover, it is important to note that it was not a clinical sample and depression and anxiety scores were low. Lastly, in the present study, we did not assess women’s prior meditation experience; therefore, it was not possible to examine how prior meditation experiences influence mindfulness profiles.

The results of the present study suggest the depth of the advantages of engaging in mindfulness-based approaches during pregnancy. In fact, a recent meta-analysis (Corbally and Wilkinson, 2021) determined that mindfulness interventions reduced symptoms of depression in perinatal women. Furthermore, the overall results of a recent systematic review (Callanan et al., 2022) found that mindfulness-based interventions were the most effective for the treatment of anxiety during pregnancy. In addition, the results of the present study indicate that the reduction in symptoms could, partially, be due to greater satisfaction with the couple’s relationship. Therefore, mindfulness interventions during pregnancy can help improve satisfaction in a couple’s relationship, which in turn is related to mitigating psychological symptoms. In this sense, midwives and psychologists in health centers have a fundamental role to play because they are the ones who monitor patients and can recommend this type of intervention.

## Data availability statement

The raw data supporting the conclusions of this article will be made available by the authors, without undue reservation.

## Ethics statement

The study involving human participants was reviewed and approved by the Clinical Research Ethics Committee of Euskadi (PI2019101) and the University of Deusto (ETK-10/19-20). The participants provided their written informed consent to participate in this study.

## Author contributions

OE-E, IO, and EC contributed to the conception and design of the study and wrote sections of the manuscript. OE-E organized the database. EC performed the statistical analysis. IO wrote the first draft of the manuscript. All authors contributed to manuscript revision and read and approved the submitted version.
